# Scattering Inversion Study for Suspended Label-Free Lymphocytes with Complex Fine Structures

**DOI:** 10.34133/2022/9867373

**Published:** 2022-11-08

**Authors:** Lu Zhang, Huijun Wang, Jianyi Liu, Shuang Chen, He Yang, Zewen Yang, Zhenxi Zhang, Hong Zhao, Li Yuan, Lifang Tian, Bo Zhong, Xiaolong Liu

**Affiliations:** ^1^Xi’an Jiaotong University, School of Mechanical Engineering, State Key Laboratory for Manufacturing Systems Engineering, Xi’an 710049, China; ^2^Xi’an Jiaotong University, Institute of Artificial Intelligence and Robotics, Xi’an 710049, China; ^3^Xi’an Jiaotong University, Key Laboratory of Biomedical Information Engineering of Ministry of Education, Xi’an 710049, China; ^4^Xi’an Jiaotong University, First Affiliated Hospital, Xi’an 710049, China; ^5^Xi’an Jiaotong University, Second Affiliated Hospital, Xi’an 710049, China; ^6^Mengchao Hepatobiliary Hospital of Fujian Medical University, The United Innovation of Mengchao Hepatobiliary Technology Key Laboratory of Fujian Province, Fuzhou 350025, China

## Abstract

*Objective and Impact Statement*. Distinguishing malignant lymphocytes from normal ones is vital in pathological examination. We proposed an inverse light scattering (ILS) method for label-free suspended lymphocytes with complex fine structures to identify their volumes for pathological state. *Introduction*. Light scattering as cell’s “fingerprint” provides valuable morphology information closely related to its biophysical states. However, the detail relationships between the morphology with complex fine structures and its scattering characters are not fully understood. *Methods*. To quantitatively inverse the volumes of membrane and nucleus as the main scatterers, clinical lymphocyte morphologies were modeled combining the Gaussian random sphere geometry algorithm by 750 reconstructed results after confocal scanning, which allowed the accurate simulation to solve ILS problem. For complex fine structures, the specificity for ILS study was firstly discussed (to our knowledge) considering the differences of not only surface roughness, posture, but also the ratio of nucleus to the cytoplasm and refractive index. *Results*. The volumes of membrane and nucleus were proved theoretically to have good linear relationship with the effective area and entropy of forward scattering images. Their specificity deviations were less than 3.5%. Then, our experimental results for microsphere and clinical leukocytes showed the Pearson product-moment correlation coefficients (PPMCC) of this linear relationship were up to 0.9830~0.9926. *Conclusion*. Our scattering inversion method could be effectively applied to identify suspended label-free lymphocytes without destructive sample pretreatments and complex experimental systems.

## 1. Introduction

Lymphocytes of human peripheral blood are an important indicator for detecting cellular immunity and humoral immunity, which is of great significance in the diagnosis of some diseases, such as malignant tumors [1], autoimmune and immune deficiency diseases [2], and blood diseases [3]. Labeling methods for lymphocyte organelles detection are widely applied for no matter traditional microscopic examination and flow cytometry [4, 5] even advanced super-resolution microscopy [6]. Unfortunately, the phototoxic and photobleaching problems in labeling detections make big challenges in maintaining the intrinsic biophysical states of cells. There are some cases that are not suitable for labeling technology. For example, it cannot be applied to the lipid [7]. Furthermore, cells growth states in the immunotherapy [8–10] should not be disturbed by any labeling processes. Because of cell’s intrinsic properties are closely related to its pathological process, the requirement that the intrinsic properties of cells can be detected without any labeling and other destructive treatments has been proposed. This problem has attracted great attention in the field of biological detections [11–14]. Light scattering is closely related to cell’s biophysical states; therefore, it is called as cell’s “fingerprint” [15]. When a light beam illuminates on a label-free cell, its spatial scattered light carries its identity characteristics (volume, structures, components, et al.). It has been proved cell’s volume change has close relationship with many malignant diseases [16–21]. For example, when patients suffer from the acute, chronic, and immature lymphocytic leukemia, the number of immature lymphocytes in blood increases with varying degrees [16, 17]. Compared with the average diameter 7.5 *μ*m of mature lymphocytes [18], immature lymphocytes increase to about 10-16 *μ*m. On the other hand, nucleus is another important indicator for cytopathologic diagnosis. It has been found that lymphocyte nuclei volumes of autism children decrease to 70% of healthy children [19], but for leukemia patients they become larger [20, 21]. Therefore, finding out the relationships between scattering features and cell’s morphology plays an important role for label-free biological detections, which is rooted in light scattering inversion (LSI) problem.

As early as 1970s, the relative volume distributions of biological particles with different diameters were estimated by one-dimensional (1D) scattering simulation method in which forward light scattering pattern (LSP) in small angle ranges was analyzed [22]. But at that time, how to detect LSP in experiments was a big challenge. LSP Fourier transform method was then put forward and experimentally verified by Semyanov et al. with the help of scanning flow cytometry [23]. In the meantime, the scattering phase function was studied to inverse biological particle sizes by a rotational confocal imaging setup [24]. Then, a further theoretical analysis in scattering inversion was done by Romanov and Yurkin [25]. However, these researches were mainly focused on 1D LSP of simple spherical particles. Su group tried to figure out the relationships of two-dimensional (2D) scattering images with biological particle sizes [26–29]. Monte Carlo algorithm was designed to simulate the distributions of spherical organelles (such as mitochondrion) inside the membrane [28, 29]. The 2D scattering speckles and fringes of label-free spherical cells in a microchannel were imaged to inverse their internal structures [30, 31]. However, spheres and ellipsoids used to approximate cells’ biological structures were quite different from their real morphologies. Up to now, the detail relationships between cell’s real morphologies and its spatial scattering are not fully understood in LSI problem. The lack of accurate and reliable inversion principles makes scattering method hard to detect label-free clinical cells.

In this study, the real morphologies with complex fine internal and external structures of clinical lymphocytes in human peripheral blood were modeled instead of normally used spheres and ellipsoids. The relationships between their 2D scattering images and volumes of membrane and nucleus in LSI problem were studied, respectively. According to our research results, we found the area values in effective boundary of forward scattering image linearly increased with cell’s membrane volumes. Although nucleus volume was normally difficult to be detected without labeling methods, its scattering image entropy calculated by the gray level cooccurrence matrix algorithm (GLCM) was proved by us to have monotonic relationship with its volume. Furthermore, LSI specificity was firstly (to our knowledge) discussed in this paper to verify the inversed relationship between scattering features and cell’s biological structures. For complex fine structures, cell’s posture, refractive index, surface fold, and the ratio nucleus to the cytoplasm were discussed in detail. In order to verify the correctness of our scattering inversion principles, scattering experiments for both standard polystyrenes microspheres and clinical leukocytes from human peripheral blood were designed. The results showed the good linear and monotonic inversion relationships between scattering features and biological structures, which proved the good ability of our method in scattering inversion for suspended label-free lymphocytes with complex fine structures.

## 2. Materials and Methods

### 2.1. Materials

#### 2.1.1. Standard Polystyrene Microspheres (SPM)

The commercial Duke polymer microspheres (Thermo Fisher Scientific Co. Ltd., USA) were used as the standard samples, which diameters, volumes, and other information are listed in Table 1.

**Table 1:** Standard polystyrene sphere parameters.

Number	Diameter (*μ*m)	Volume (*μ*m^3^)	Original concentration (per ml)	Std. dev (*μ*m)	C.V
1	4.000±0.043	33.51	$5\times 10^{7}$	0.021	1.0%
2	5.021±0.041	66.28	$1\times 10^{7}$	0.050	1.0%
3	6.007±0.040	113.49	$1\times 10^{7}$	0.060	1.0%
4	6.982±0.045	178.21	$1\times 10^{7}$	0.070	1.0%

Std. dev: standard deviation; C.V: coefficient of variance.

#### 2.1.2. Lymphocytes in Human Peripheral Blood

Fresh human peripheral blood (2 ml) from healthy volunteer was collected in 5 ml EDTA vacutainer tube (Shijiazhuang Kangweishi Medical Equipment Co. Ltd., China). The blood was diluted by 2 ml phosphate buffer saline (PBS, pH 7.4) and then mixed with 4 ml lymphocyte separation medium (LSM, Tian Jing Haoyang Biological Manufacture Co. Ltd., China). The mixture was layered after centrifugation (400 g for 25 minutes) at room temperature. From the top to bottom of the layered mixture, there were plasma, lymphocytes, LSM, and erythrocytes. The layered lymphocytes were extracted by the pipette and washed with 5 ml PBS. After the second centrifugation (400 g for 5 minutes), lymphocytes gathered at the bottom were obtained by removing the supernatant fraction. Normally, the diameters of the obtained lymphocytes range from 4.8 to 12.0 *μ*m [18] with the average value 7.5 *μ*m.

### 2.2. Methods

**Figure 1:**
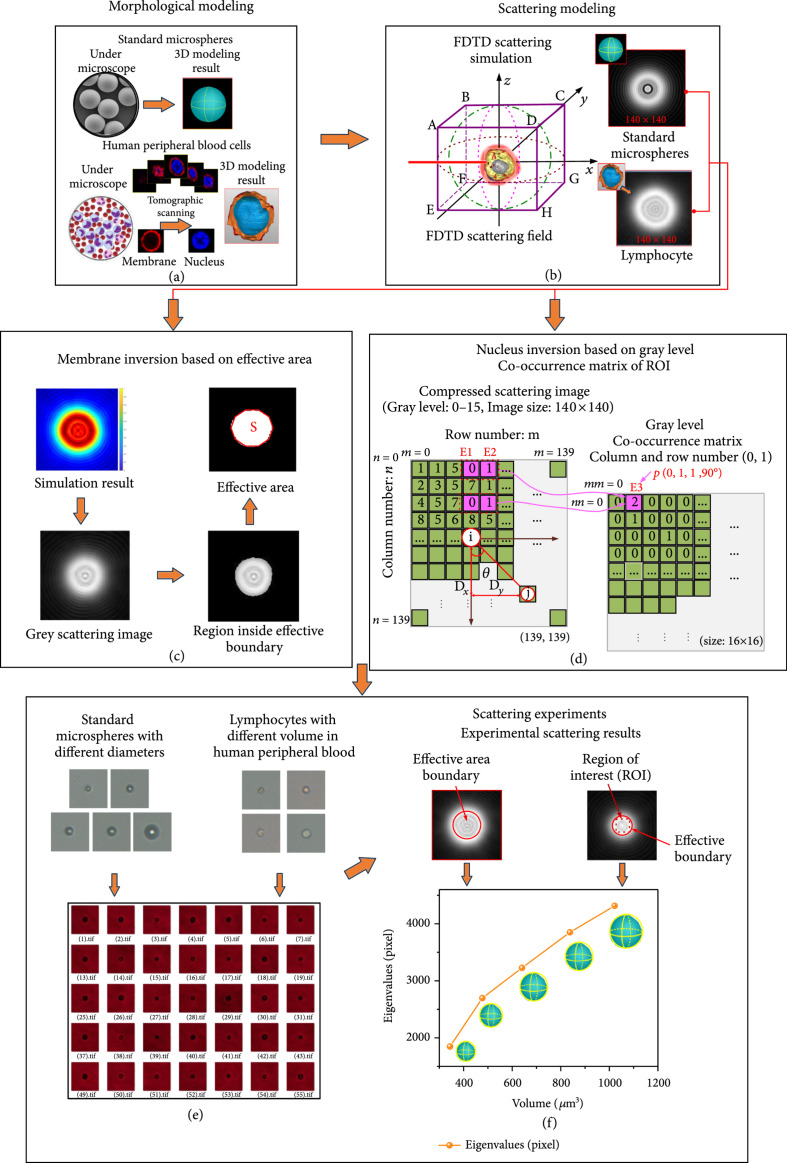
Simulation and experimental methods: (a) morphological modeling, (b) scattering modeling, (c) membrane inversion method, (d) nucleus inversion method, (e) experimental scattering images, and (f) inversion principle verification.

Label-free lymphocyte scattering was studied to inverse the volumes of their membrane and nucleus, which is explained in Figure 1. As shown in Figures 1(a) and 1(b), morphological modeling and light scattering modeling should be carried out, respectively. Then, the area inside effective boundary of simulated forward scattering images and GLCM’s entropy of the region of interest (ROI) were calculated as two important parameters to inverse theoretically the volumes of label-free cells’ membranes and nuclei, as shown in Figures 1(c) and 1(d). In order to verify the theoretical inversion principles, forward scattering experiments for SPM and lymphocytes were designed, and scattering images were recorded as Figure 1(e). The inversion principles in Figure 1(f) were obtained. Finally, the experimental results were compared with theoretical inversion principles to assess its reliability and correctness.

#### 2.2.1. Morphology Modeling

In order to study the inversion principles of suspended label-free lymphocytes with complex fine structures, 3D morphology modeling was very necessary. Different from traditional modeling methods which applied spheres and ellipsoids to represent the internal and external cellular structures, in this study clinical lymphocyte morphologies in suspension were detected layer by layer firstly by fluorescence confocal microscope (FCM). Their 3D morphologies of membranes and nuclei were reconstructed by our methods published before [32, 33]. The membrane and nucleus morphologies of clinical lymphocytes then were obtained as the basic morphology models. To generate different fine structures, the Gaussian random sphere geometry algorithm was introduced herein to make basic morphologies have complex fine structures, such as the fold and roughness on surface. The Gaussian random sphere geometry algorithm could generate a statistically related class for irregularly shaped objects, which had been used to model small objects, such as ice crystals [34], sand particles [35], and comets [36]. To our best knowledge, it seldomly applied in biological objects.

Complex fine structures generated by Gaussian random sphere geometry algorithm can be described as covariance function with logarithmic radius. A basic lymphocyte model is divided into N discrete points. In spherical coordinate, their radii $r_{N}\left(\theta ,\varphi \right)$ are modeled by Gaussian random sphere geometry algorithm, by which the radius of each discrete point follows the multivariate distribution.

$r_{N}\left(\theta ,\varphi \right)$ in spherical coordinate has the relationship with its logarithmic radius $s_{N}\left(\theta ,\varphi \right)$ as follows:

(1)rNθ,φ=r¯1+σ2expsNθ,φ,

wherein $\bar{r}$ is their mean radius. σ is the radius relative standard deviation. The logarithmic radii for all N discrete points can be expressed as $s={\left(s_{1},\cdots ,s_{N}\right)}^{\text{T}}$. Corresponding to the given spherical coordinate, s satisfies the multivariate normal statistical distribution:

(2)nNs,∑S=12πNdet∑Sexp−12sT∑S−1s,

where $\sum^{s}$ is defined as the covariance matrix of logarithmic radii where the superscript s represents the logarithmic radii. And $\sum^{s}$ can be expanded into the following equation:

(3)∑Sγ=lnσ2+1CSγ=lnσ2+1∑l=lminl=lmaxl−νPlcosγ,

wherein γ is the angular distance between two directions $\left(\theta_{1},\varphi_{1}\right)$ and $\left(\theta 2,\varphi 2\right)$, and $C_{S}\left(\gamma \right)$ is the correlation function for logarithmic radius. $C_{S}\left(\gamma \right)$ can be expanded with a Legendre series $P_{l}$. The Legendre series has been truncated from the lowest order $l_{\min}$ to the highest order $l_{\max}$. Considering the modeling accuracy and simulation time, $l_{\min}$ and $l_{\max}$ are set to 2 and 50. The superscript ν is the power law coefficient. According to Equations (1)–(3), σ, ν, and $\bar{r}$ are important to determine the radii $r_{N}\left(\theta ,\varphi \right)$ for complex fine structures. σ controls the fluctuation degree, and ν controls the number of fold fluctuations in unit solid angle. The parameter ξ (ξ=σ+1/ν) is selected to describe the finally generated fine structure features on 3D basic morphology models.

#### 2.2.2. Scattering Modeling

**Figure 2:**
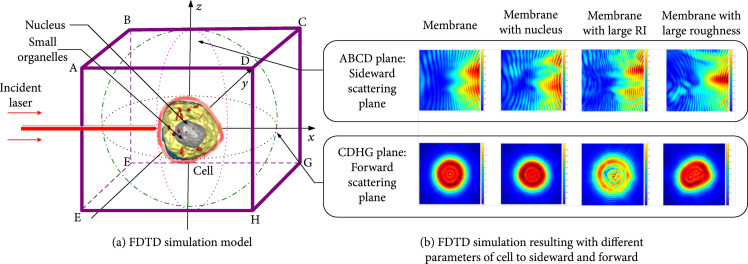
Forward and sideward scattering results by FDTD simulation.

FDTD algorithm is based on the finite differences instead of differentials to solve Maxwell’s equations, which was selected in this study to simulate the scattering distributions of lymphocytes in space. According to our former researches [37], in scattering modeling, the illuminating incident light was set with the wavelength $\lambda_{0}=632.8\;\text{nm}$ along the forward direction of x axis as shown in Figure 2. The amplitude normalization was set to 1. Cell nucleus and membrane were located in the position of coordinate origin to form the standard nucleus-membrane double-layer model. Small organelles (mitochondrion) were located based on the reconstructed results by above morphology modeling method. As shown in Figure 2(a), the far field scattering receiver was built as a cube. The scattering receiver plane CDHG was the forward scattering plane which was used in our scattering inversion, and ABCD was the sideward scattering plane. The perfectly matched layer boundary condition (PML) was adopted and in order to guarantee the correctness and accuracy. The simulating calculation step size was set as λ/10. Figure 2(b) presents the forward and sideward scattering modeling results for lymphocyte models with different structures.

**Figure 3:**
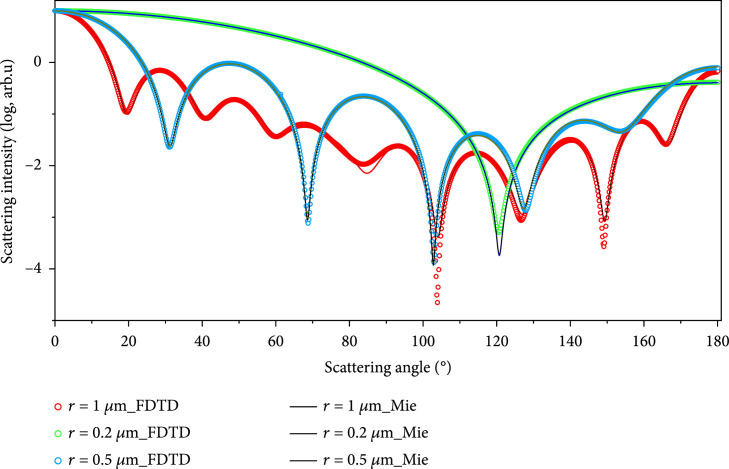
FDTD simulation verification by Mie theory.

In order to verify the correctness of our programs and parameter setting, the LSPs of microspheres with radius of 0.2 *μ*m, 0.5 *μ*m, and 1.0 *μ*m calculated by our FDTD were compared with the results of Mie theory (classical algorithm for solving spherical media scattering), which is shown in Figure 3. It could be seen that LSPs of our FDTD method were in good agreement with classical Mie simulation. The relative errors for different microspheres were all within 0.92%, which confirmed the reliability and accuracy of our FDTD programs.

#### 2.2.3. Experimental System

**Figure 4:**
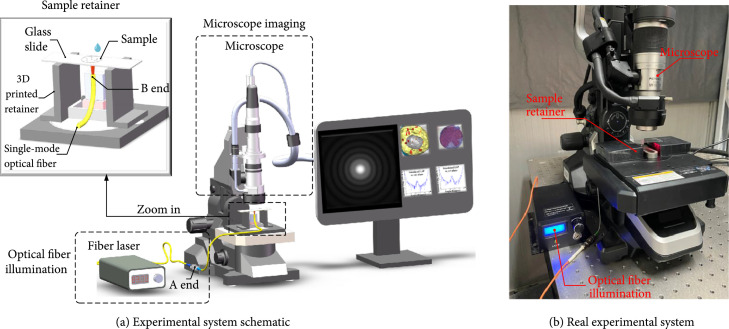
Experimental system.

Experimental system was consisted of three parts: the illumination section by optical fiber laser, the scattering imaging section by microscope, and the sample fixing section by self-designed retainer, as shown in Figure 4(a). The single-mode optical fiber (Changfei Optical Fiber and Cable Co. Ltd., NA=0.124, China) was selected to transmit incident light onto a single lymphocyte. One end (A end in Figure 4(a)) of the single-mode optical fiber coupled with the fiber laser which wavelength and power are 650 nm and 2 mW, respectively (Beijing Xinglin Ruiguang Technology Co. Ltd., China). After polishing, the other end (B end) of the fiber was fixed by the sample retainer and faced perpendicularly to the bottom of glass slide. A drop of lymphocyte solution was dripped onto the grass slide which was fixed on the top of a 3D displacement table. In our experiments, the distance from B end to the bottom of the glass slide was about 12 mm. Glass slide, the retainer, single-mode optical fiber, and the 3D displacement table were all put on microscope stage. During experiments, a single lymphocyte was firstly observed clearly in bright field without oil immersion. Then, turn on the laser and adjust B end carefully to illuminate only a single lymphocyte. After turning off the bright field, forward scattering images (size: 140×140 pixel) were collected on defocus plane (defocus distance about 30 micrometers). By image processing, the scattering features for scattering inversion principles could finally be obtained.

#### 2.2.4. Image Processing

The effective area in forward scattering image was used for scattering inversion of membrane volume. The pixels whose gray values were about 57% of the maximum were defined as effective boundary. The area inside the effective boundary was the effective area. For scattering inversion of nucleus volume, GLCM algorithm was put forward to calculate the eigenvalues of ROI. GLCM is the matrix [38, 39] which row and column numbers are equal to the number of image grayscale levels. Its matrix element $P\;\left(i,j,d,\theta \right)$ represents the occurrence probability of pixel pairs with gray values i and j. For GLCM, there are three key parameters g, d, and θ. g is the original image gray level. d is the distance between pixel pairs when GLCM is generated, which equals to 1, 2, 3, 4, 5…. And θ indicates the positional relationship of pixel pairs, usually selected as 0°, 45°, 90°, and 135°. The grayscale of our scattering image is g=256. In order to reduce calculation time, the grayscale of scattering image should be compressed but without losing many important details. The gray level can be compressed to 16, 32, 64, and 128. Their computational times of extracting eigenvalues are shown in Table 2. Considering the computational efficiency, the gray level g was finally determined to be 16, and d equaled to 1. To ensure the changes of image texture in all directions were included, the final eigenvalues were the average values in the four directions of 0°, 45°, 90°, and 135°. In our experiments, four commonly used eigenvalues in describing texture features were selected:

(4)ASM=∑i=0g−1∑j=0g−1P2i,j,d,θ,Entropy=−∑i=0g−1∑j=0g−1Pi,j,d,θ⋅logPi,j,d,θ,Contrast=∑i=0g−1∑j=0g−1i−j2Pi,j,d,θ,IDM=∑i=0g−1∑j=0g−111+i−j2Pi,j,d,θ.

**Table 2:** The processing time of images with different gray levels.

Gray level	16	32	64	128

Time (s)	5.26	19.01	74.36	289.00

## 3. Results and Discussion

### 3.1. 3D Modeling Results for Lymphocytes with Complex Fine Structures

**Figure 5:**
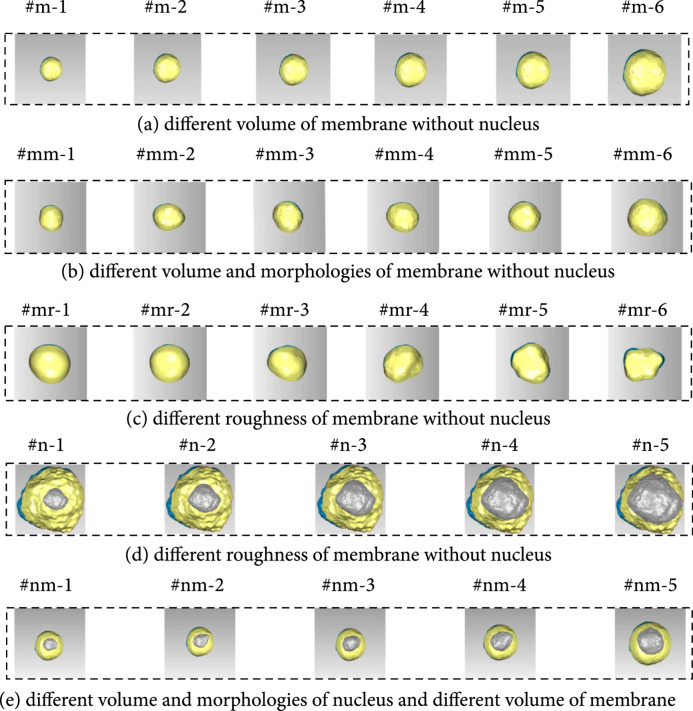
Lymphocyte morphology models with complex fine structures.

750 lymphocytes in suspension from fresh human peripheral blood were detected by fluorescence confocal microscope. 3D lymphocyte models with five groups of different complex fine structures were constructed, which included the models with different membrane volumes but without nucleus (as shown in Figure 5(a)), the models with both different volumes and morphologies of membrane but without nucleus (as shown in Figure 5(b)), the models with different roughness on membrane’s surface but without nucleus (as shown in Figure 5(c)), the models with different nucleus volumes but with the same membranes (as shown in Figure 5(d)), and the models with both different volumes and morphologies but with different membrane volumes (as shown in Figure 5(e)).

### 3.2. Simulated Scattering Inversion Principles

#### 3.2.1. Membranes with Different Volumes

**Figure 6:**
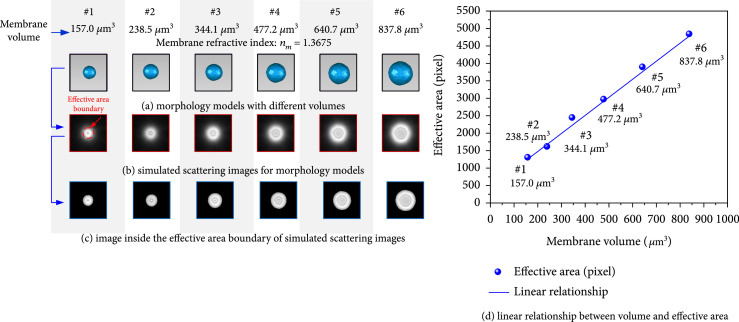
Relationship between different lymphology membrane volumes and effective areas.

Lymphocyte membrane models with the same surface folds but without nucleus were built, and their volumes were 157.0 *μ*m^3^, 238.5 *μ*m^3^, 344.1 *μ*m^3^, 477.2 *μ*m^3^, 640.7 *μ*m^3^, and 837.8 *μ*m^3^, respectively, as shown in Figure 6(a). Their membrane refractive index was set as 1.3675, and the membrane roughness ξ was 0.48. The simulated forward scattering results are shown in Figure 6(b). The effective boundaries in Figure 6(b) are extracted and shown in Figure 6(c). After calculating effective area, a good linear relationship with the increase of lymphocyte membrane volumes was found (seeing Figure 6(d)). In order to evaluate its linear correlation, the commonly used parameter PPMCC was selected. For perfect negative and positive correlation, PPMCC equals to -1 and +1, but for no correlation, it equals to 0. In Figure 6(d), PPMCC for our scattering inversion was up to 0.9979, which meant lymphocyte membrane volume and its effective area in forward scattering image had a good linear inversed principle.

**Figure 7:**
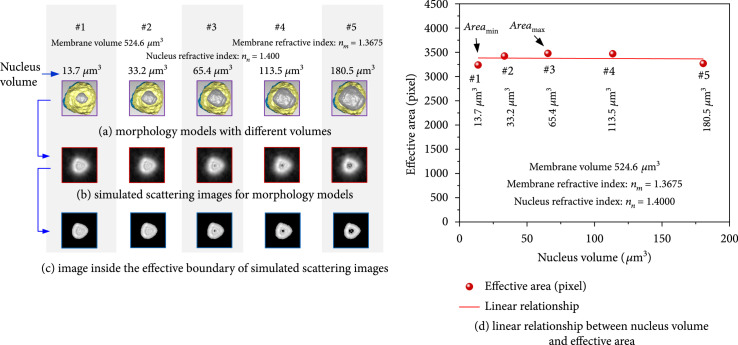
Influence of the nucleus for relationship between membrane volume and effective area.

To evaluate the specificity of this linear relationship, some other biometric differences for lymphocyte complex fine structures were considered. Because human lymphocytes have a large ratio of nucleus to the cytoplasm, the influence of the nuclei on this linear inversion principle should be firstly discussed. A control group with five lymphocyte models had the same membrane volume (524.6 *μ*m^3^) and refractive index (1.3675), but different nucleus volumes were set up in Figure 7(a). Their fold roughness was kept unchanged. The simulated forward scattering images and processed effective boundaries are shown in Figures 7(b) and 7(c). The effective area is then plotted in Figure 7(d). It could be known that the relationship between effective area and nucleus volumes was almost unchanged as displaying in Figure 7(d) like a horizontal line. And for different nucleus volumes, the effective area changed only less than 3.1%. It meant that the effective area had good specificity for membrane volume in scattering inversion.

**Figure 8:**
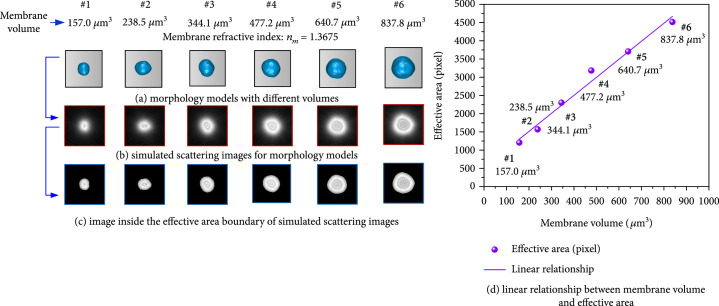
Specificity analysis for linear relationship with controlled volumes but random morphologies.

Since suspended living lymphocytes always change their postures, the influence of postures needs to be considered. A group of models with randomly generated morphologies but controlled volumes in Figure 8 were discussed. It could be seen in Figure 8(d) that their relationship between membrane volume and its effective area in forward scattering image was still linear, and PPMCC for Figure 8(d) was calculated to be 0.9911. So, it proved the inversion principle was not affected by different postures, and the effective area was well specific to membrane volume.

**Figure 9:**
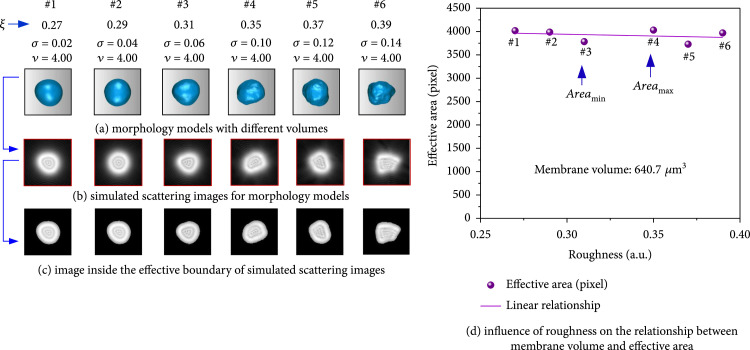
Influence of membrane surface roughness on the effective area of forward scattering images.

On the other hand, the influence of membrane surface fold on the specificity of this scattering inversion principle was considered. Lymphocyte models with different membrane roughness ξ are set up in Figure 9(a). Their simulated scattering images and effective area results are given in Figures 9(b) and 9(c). The deviation $\delta_{\text{v}}$ is defined as $\delta_{\text{v}}=\left(\left({\text{Area}}_{\max}-{\text{Area}}_{\min}\right)/{\text{Area}}_{\max}\right)\times 100\%$ was selected to quantitatively evaluate the influence on the specificity. Area in this equation stood for the effective area, and v was the membrane volume. According to the results in Figure 9(d), the influence of surface fold was not obvious, and the deviation $\delta_{\text{v}}$ between the maximum ${\text{Area}}_{\max}$ and the minimum ${Area}_{\min}$ was less than 3.5%.

Therefore, the conclusion could be drawn that the effective area of forward scattering image was the specific parameter to identify membrane volumes of label-free lymphocytes in suspension.

#### 3.2.2. Nucleus with Different Volumes

**Figure 10:**
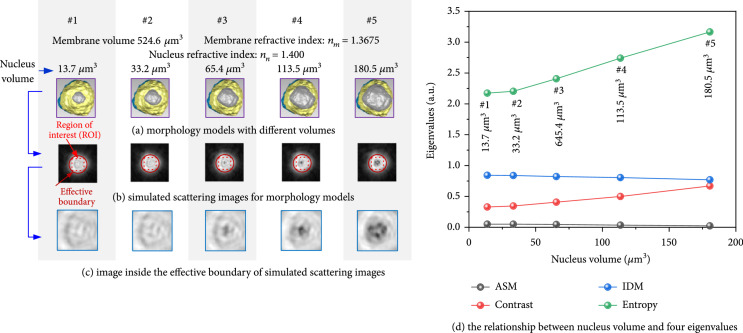
The relationship between nucleus volume and four eigenvalues.

To discuss the inversion principle for nucleus from scattering images, five lymphocyte models are set up in Figure 10(a) with different nucleus volumes but the same membrane volume (524.6 *μ*m^3^), refractive index (1.3675 for membrane and 1.4000 for nucleus), and surface folds (0.48 for membrane and 0.41 for nucleus). Their nucleus volumes were 13.7 *μ*m^3^, 33.2 *μ*m^3^, 65.4 *μ*m^3^, 113.5 *μ*m^3^, and 180.5 *μ*m^3^, respectively. ROI (in the red dot squares of Figure 10(b)) was an inner knot square region inside the effective boundary. Their zoomed-in images of ROI are shown in Figure 10(c). The typical four GLCM characters of ROI, angular second moment (ASM), contrast, inverse difference moment (IDM), and entropy, are given out in Figure 10(d). As mentioned in Section 3.2.1, although the effective area of forward scattering image was not sensitive to nucleus volumes, the entropy of ROI in Figure 10(d) increased with nucleus volume, and they had a good linear relationship.

**Figure 11:**
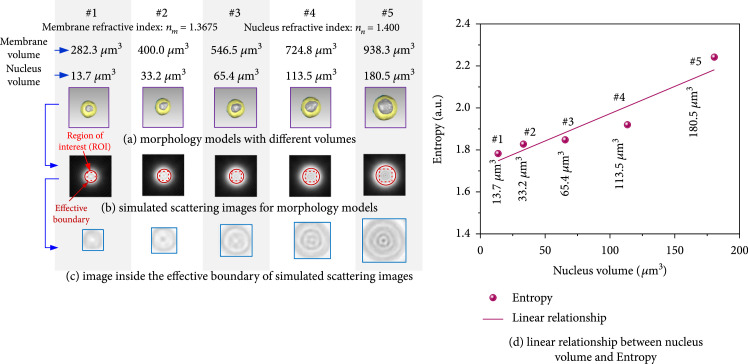
The linear relationship between the entropy and nucleus volume.

In order to evaluate the specificity of nucleus volume and the entropy, lymphocyte models with both different volumes and various nucleus morphologies are set up in Figure 11(a). Their extracted ROIs and zoomed-in results are given out in Figure 11(b) with the red dot squares and Figure 11(c). Because the membrane volumes in this group were different, their ROI pixel sizes varied from $23\times 23\;\text{pixe}{\text{l}}^{2}$, $28\times 28\;\text{pixe}{\text{l}}^{2}$, $35\times 35\;\text{pixe}{\text{l}}^{2}$, $40\times 40\;\text{pixe}{\text{l}}^{2}$, and $50\times 50\;\text{pixe}{\text{l}}^{2}$, respectively. And their GLCM entropy results are calculated and then plotted in Figure 11(d). Although in this group of lymphocyte models the membrane volumes changed, the linear monotonic relationship between nucleus volume and ROI entropy was still kept. PPMCC for their linear relationship was up to 0.9452. Therefore, ROI entropy could be determined as the specific parameter to inverse nucleus volumes of label-free lymphocytes in suspension.

#### 3.2.3. Organelles with Different Refractive Indexes

**Figure 12:**
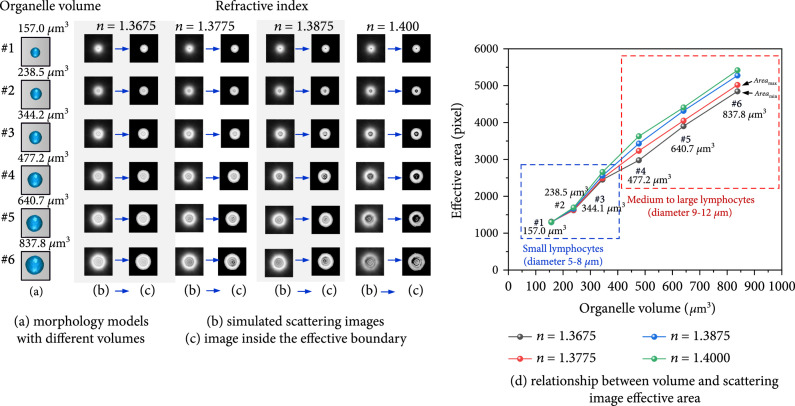
The specificity analysis for linear inversion principle with different refractive indexes.

Leukocytes are composed of various organelles. Besides their membranes and nuclei, organelles with different refractive indexes should also be considered. A group of organelles with different refractive indexes and volumes is modeled in Figure 12(a). Their refractive indexes were set as 1.3675, 1.3775, 1.3875, and 1.400, respectively, and the surface roughness (ξ=0.48) and posture were the same. After FDTD simulation and image processing, their forward scattering images and effective boundary are obtained in Figures 12(b) and 12(c), and effective area values are plotted in Figure 12(d). According to the clinical statistics information, the models #1 to #3 with diameter 5~8 *μ*m had the similar size to human small lymphocytes, and the models #4 to #6 as medium to large lymphocytes. In Figure 12(d), it could be known that the refractive index had less influence on effective area of the models #1 to #3 in the blue box with small volumes. Such as the model #1 (membrane volume: $157.0\mu {\text{m}}^{3}$), for different refractive indexes in Figure 12(d), its effective areas were almost the same. In fact, except membrane and nucleus, no organelles could have such large volume because their diameters were only several hundreds of nanometers. Therefore, the influence of refractive index in scattering inversion could be ignored for a single organelle. Even when the organelles like mitochondria gathered as a group due to their different distributions, the whole volume was hard to be equal to that of model #1. Another case was the aggregation of several nuclei. Under this circumstance, their whole volume became large, but it was still less than that of model #6 which influence of refractive index on effective area could be seen as shown in the red box of Figure 12(d). The volume of the model #4 to #6 was similar to that of medium and large human lymphocytes. In order to assess the influence of refractive indexes on effective area for the model #4 to #6, their deviations $\delta_{\text{v}}$ were calculated. As a result, the maximum $\delta_{\text{v}}$ of four different refractive indexes were still less than 5.0%. It showed that on the one hand, effective area was not sensitive to refractive index for both larger membrane, nucleus, and small organelles, and on the other hand, its specificity for membrane volume was proved indirectly.

In fact, the components of human lymphocyte organelles are mainly lipids and proteins, whose refractive indices are about 1.36~1.37, such as the black line (n=1.3675) and the red line (n=1.3775) in Figure 12(d). Compared their area values on the red and black lines, their maximum $\delta_{\text{v}}$ for the model #1 to #6 was also less than 3.4%. So, it could be known that the difference on refractive index had little effect on scattering inversion between organelle volume and effective area.

### 3.3. Experimental Verification for Inversion Principle

Two kinds of samples were selected to verify the scattering inversion principles. The first one was the SPM in suspension with known morphology and diameter, which could quantitatively assess the precision of our scattering inversion principles. And the second one was the clinical label-free lymphocyte in vivo from human peripheral blood by which the effectiveness of scattering inversion principles in practical applications could be verified.

**Figure 13:**
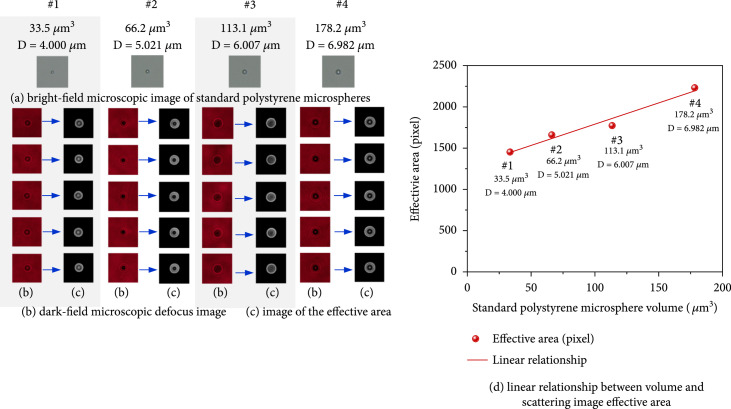
Linear relationship between the effective area and standard polystyrene microsphere volume.

**Figure 14:**
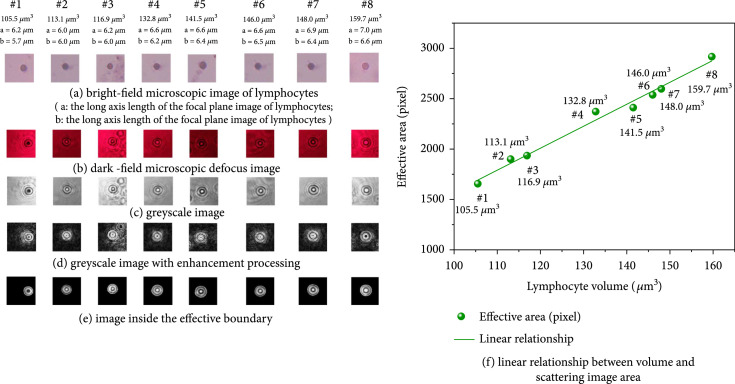
Linear relationship between the effective area and lymphocyte volume.

Suspended SPMs in Figure 13(a) (microscope observed) with diameters of 4.000 *μ*m, 5.021 *μ*m, 6.007 *μ*m, and 6.982 *μ*m (corresponding volumes: 33.5 *μ*m^3^, 66.2 *μ*m^3^, 113.1 *μ*m^3^, and 178.2 *μ*m^3^) were adopted for scattering imaging experiments, and their forward scattering images (in Figure 13(b)) were captured five times. The average effective area results (in Figure 13(c)) were calculated. The relationship between SPM volume and average effective area is shown in Figure 13(d). Eight sphere-like lymphocytes from human peripheral blood were also imaged. Their morphologies under microscopic observation are shown in Figure 14(a). Since the volumes of lymphocytes were unknown, so the cell was thought as an ellipsoid formed by the rotation of the focal plane image (Figure 14(a)) about axis a, where a and b were the major and minor axis lengths on the focusing plane, respectively. As a result, the calculation equation of elliptical volume $V=\left(\pi /6\right){ab}^{2}$ was applied to approximate that of lymphocytes. The edge enhancement process is done and shown in Figure 14(d) due to the low contrast between effective boundary and the background. Then, effective area values in Figure 14(e) are calculated and plotted in Figure 14(f). It could be seen from Figure 13(d) and Figure 14(f) that there were good linear relationships between the volume and effective area for both SPMs and clinical lymphocytes. The PPMCC was up to 0.9830 for SPM and 0.9926 for lymphocytes. Therefore, the scattering inversion principles concluded in Section 3.2 for suspended label-free lymphocytes with complex fine structures were well approved.

## 4. Conclusions

In this study, we proposed a method for inversing membrane and nucleus volume of label-free lymphocytes in suspension. It had been proved that the effective area in forward scattering image is closely related to its membrane volume, which was not sensitive to the surface roughness, the posture, and the ratio of nucleus to the cytoplasm and refractive index. And the entropy of ROI in forward scattering image had the ability to inverse nucleus volume, which was normally difficult to be detected without labeling method. The scattering image inversion principles in this study could be easily applied to identify suspended label-free lymphocytes in human peripheral blood, and the viability and intrinsic biological characters of cells could be well preserved. Our method also could be extended to other kinds of cells. On the other hand, our experimental system simply consisted of an ordinary microscope and a fiber laser. Compared with the labeling detection methods, it reduced the complexity of experimental system and sample pretreatments. In the further study, this method could be combined with machine learning algorithms to directly identify pathological lymphocytes from normal ones based on the volume differences of membrane and nucleus. And different kinds of microfluidic chips can be easily integrated into our experimental system, by which both the identification and isolation of label-free cells will become rapid and simple.

## Data Availability

The data of scattering images to support the results of this study are available on request from the corresponding authors.
